# World Vitiligo Day: lessons from Mexico's annual headquarters and its real-world impact

**DOI:** 10.3389/fmed.2024.1502924

**Published:** 2024-11-14

**Authors:** Jorge Ocampo-Candiani, Julia Sigova, Torello Lotti, Yan Valle

**Affiliations:** ^1^Hospital Universitario Dr. José e, Universidad Autónoma de Nuevo León, González, Mexico; ^2^Department of Neonatology, Faculty of Continued Medical Education, Pirogov Russian National Research Medical University, Moscow, Russia; ^3^Department of Dermatology, University of Studies Guglielmo Marconi, Rome, Italy; ^4^Vitiligo Research Foundation, New York, NY, United States

**Keywords:** vitiligo, awareness, patient support, advocacy, ethnic populations, Google Trends

## 1 Introduction

Vitiligo has been known since ancient times as a depigmentation disorder that severely impacts quality of life (11). This autoimmune condition, caused by the loss of melanocytes and resulting in white patches on the skin, is relatively common and straightforward to diagnose. However, despite recent advances in understanding its pathogenesis and refining management strategies, effective treatments remain elusive (12).

We read with great interest the article by Juntongjin et al. (1) on the awareness of vitiligo among multi-ethnic populations visiting a private hospital in Bangkok, Thailand. The authors found that individuals of Arab descent had the highest knowledge scores, while those of European origin exhibited the most positive attitudes. In response to their conclusion that “global awareness of vitiligo should be emphasized,” we would like to share our experience with the World Vitiligo Day (WVD) annual campaign headquarters in Mexico and highlight its impact both online and offline, offering it as a potential model for future events.

## 2 World Vitiligo Day: a global initiative

### 2.1 Evolution and recognition

Since its modest beginning as a 3-h inauguration event at the University of Guglielmo Marconi (Rome, Italy) in 2012, WVD has grown into an annual, global 3-day celebration (2). Thanks to community leaders such as Valarie Molyneaux from VITFriends and countless others, the campaign has received official recognition from over 20 U.S. city mayors and state governors, including the most recent by the General Assembly of Pennsylvania State House (3).

In 2019, the rolling WVD headquarters were set in Hanoi, Vietnam, where the event gained formal recognition from the Vietnamese government—a significant milestone in its regional impact, which we hypothesize may have also influenced vitiligo awareness in Thailand (4).

### 2.2 Mexico: empowering awareness and education

The 12th edition of WVD, hosted in Mexico in 2022, exemplified the campaign's influence on vitiligo awareness and education. Under the theme “Learning to Live with Vitiligo,” the event was led by Drs. Jorge Ocampo-Candiani and Rossana Llergo Valdez, with key contributions from Drs. Judith Domínguez Cherit, José Alberto Ramos Garibay, Abraham Alfaro García, and Karen Férez Blando. This event went beyond awareness-raising and significantly enhanced education for both patients and physicians.

**Figure 1:**
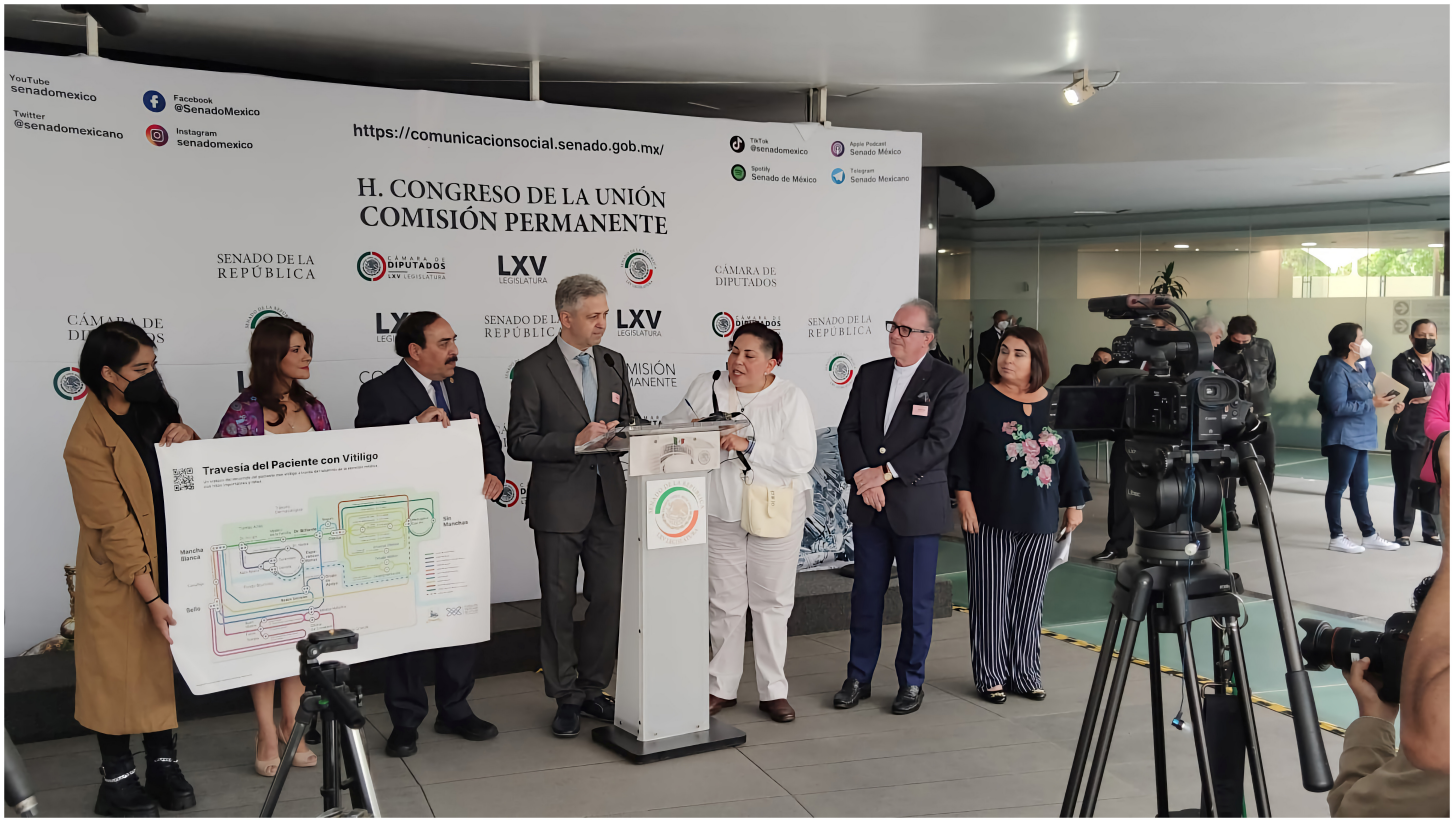
WVD opening ceremony at the Senate of Mexico was broadcast live on Parliament TV. From **left** to **right**: (name of assistant not recorded), Karen Férez, Jorge Ocampo-Candiani, Yan Valle, Garcia Morena, Torello Lotti, and Antares Vázquez Alatorre. Image provided by Julia Sigova.

On June 22, the WVD event opened with a press conference in the Senate's Federalism Courtyard, broadcast live on Parliament TV (Figure 1). Senator Antares Vázquez Alatorre emphasized the need for Mexico to improve medical care and establish policies to enhance the visibility and understanding of skin conditions.

The Scientific Session included contributions from 21 local clinicians and international experts over the next 2 days. Dr. Torello Lotti presented the latest advancements in vitiligo treatment, advocating a three-step approach centered on awareness, education, and professional care. Yan Valle introduced the “Vitiligo Patient Journey Map,” a tool designed to help patients and doctors navigate both treatment and non-treatment options more effectively (13).

On June 25, the formal date of World Vitiligo Day, a Patient Information Session was held at the Instituto Nacional de Ciencias Médicas y Nutrición. Workshops addressed vitiligo's impact on childhood, sexuality, work, and aesthetics, including a self-makeup session. A photographic exhibition titled “Vitiligo: A Colorful Skin” celebrated the beauty and diversity of vitiligo in the National Science Institute's exhibition hall. This patient-centered approach aligns with Juntongjin et al.'s findings on the importance of addressing misconceptions and fostering positive attitudes toward vitiligo.

### 2.3 Real-world impact and outcomes

The lasting impact of WVD 2022 in Mexico is evident in the growth of the Mexican Vitiligo Foundation (MVF), led by Dr. Karen Férez. While establishing and maintaining a local support community for vitiligo patients can be challenging, it is highly rewarding (5). Since 2022, the MVF has been particularly successful in helping individuals—especially those outside the healthcare system—come to terms with their condition and improve their quality of life through education, workshops, and creative projects.

Evidence supports the benefits of peer support provided by “promotores de salud” (community health workers) (6). Google Trends data further illustrate the campaign's impact: a significant spike in vitiligo-related searches followed WVD 2022 in Mexico, highlighting increased awareness and engagement. Although Google Trends provides only relative search volume, not absolute values, it allows for comparisons between dermatological conditions (7). Unlike melanoma or atopic dermatitis, which experienced cyclical search trends with modest growth over the last 3 years, vitiligo-related searches surged notably after the WVD event.

## 3 Discussion

The success of the WVD campaign, particularly the 12th edition in Mexico, underscores the effectiveness of targeted awareness initiatives in dermatology (8). These outcomes reinforce the importance of focused efforts in education and awareness, addressing the gaps identified by Juntongjin et al. in multi-ethnic populations.

This strategy has repeatedly proven to be the most effective path forward across several healthcare domains (9, 10). When the local groundwork is already laid, WVD acts as a catalyst, propelling awareness efforts and supporting the scientific and medical advancements happening on the ground. It is this combination of global reach and local activation that drives the campaign's success and, in turn, increases vitiligo awareness among ethnic communities.

Given the dynamic nature of awareness campaigns, both in-person and online, future studies should use statistical techniques such as controlled experiments, data normalization, and segmented analysis. These methods will help collect detailed insights, mitigate potential biases, and assess shifts in user awareness and behavior following the next round of the WVD campaign.
